# Evolution of a bispecific G-quadruplex-forming circular aptamer to block IL-6/sIL-6R interaction for inflammation inhibition†

**DOI:** 10.1039/d4sc02183e

**Published:** 2024-07-17

**Authors:** Lili Yao, Lei Wang, Shuai Liu, Hao Qu, Yu Mao, Yingfu Li, Lei Zheng

**Affiliations:** a School of Food and Biological Engineering, Hefei University of Technology Hefei 230009 China maoyuyu212@163.com lzheng@hfut.edu.cn; b Department of Biochemistry and Biomedical Sciences, McMaster University Hamilton L8S4K1 Canada liying@mcmaster.ca

## Abstract

IL-6 (interleukin-6) is an essential cytokine that participates in many inflammatory and immune responses, and disrupting the interaction between IL-6 and its receptor sIL-6R (soluble form of IL-6 receptor) represents a promising treatment strategy for inflammation and related diseases. Herein we report the first-ever effort of evolving a bispecific circular aptamer, named CIL-6A6-1, that is capable of binding both IL-6 and sIL-6R with nanomolar affinities and is stable in serum for more than 48 hours. CIL-6A6-1 can effectively block the IL-6/sIL-6R interaction and significantly inhibit cell inflammation. Most importantly, this bispecific aptamer is much more effective than aptamers that bind IL-6 and sIL-6R alone as well as tocilizumab, a commercially available humanized monoclonal antibody against sIL-6R, highlighting the advantage of selecting bispecific circular aptamers as molecular tools for anti-inflammation therapy. Interestingly, CIL-6A6-1 is predicted to adopt a unique structural fold with two G-quadruplex motifs capped by a long single-stranded region, which differs from all known DNA aptamers. This unique structural fold may also contribute to its excellent functionality and high stability in biological complex media. We anticipate that our study will represent a significant step forward towards demonstrating the practical utility of bispecific DNA aptamers for therapeutic applications.

## Abstract 2

We report a circular DNA aptamer isolated from a synthetic DNA library to bind both IL-6 and sIL-6R with nanomolar affinities. This aptamer can effectively block the IL-6/sIL-6R interaction and significantly inhibit cell inflammation.

## Graphical abstract

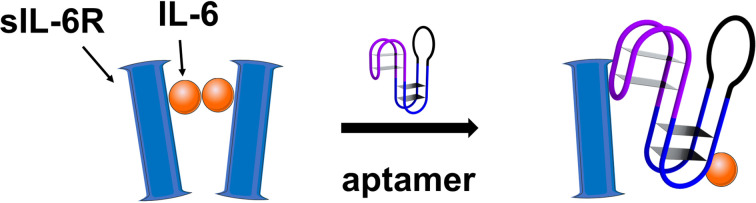

## Introduction

Interleukin-6 (IL-6) is a multifunctional cytokine, which plays a key role in many inflammatory and immune diseases, such as rheumatoid arthritis, asthma, or Crohn's disease.^1^ IL-6 interacts with the soluble form of IL-6 receptor (sIL-6R), a key molecule in the *trans*-signalling pathway that regulates pro-inflammatory reactions.^2–4^ Blocking IL-6/sIL-6R interaction has been shown to be a promising therapeutic strategy for inhibiting various inflammatory and immune diseases.^5–7^ It is worth mentioning that IL-6/sIL-6R interaction has also been reported to be involved in the progression of nonautoimmune diseases including cytokine storm in coronavirus disease 2019 (COVID-19). Tocilizumab, an immunosuppressive monoclonal antibody targeting sIL-6R, has been used for the treatment of severe COVID-19 patients, since it can block the IL-6/sIL-6R interaction and alleviate the cytokine release syndrome.^8–10^

However, monoclonal antibody-based therapy often suffers from inherent disadvantages such as high production cost and high frequency of immune-related adverse events. In contrast, aptamers, which can be obtained by the SELEX technique from synthetic random-sequence DNA libraries, can be easily synthesized and are considered non-immunogenic.^11–15^ Therefore, aptamers have great potential as antibody substitutes for therapeutic applications.^16,17^ However, natural linear aptamers are vulnerable to nuclease degradation in biological media and are susceptible to the influence of large amount of irrelevant substances in biological media because of their conformational flexibility, which can seriously hinder the practical therapeutic application of aptamers.^18,19^ Recently, circular aptamers have aroused a lot of interest because of their enhanced biostability and reduced conformational flexibility.^20–22^ For example, Daniela *et al.* have constructed circular thrombin aptamers with enhanced biological stability from linear ones.^23,24^ Liu *et al.* have directly selected circular aptamers from a circular DNA library and shown that thus derived circular aptamers targeting glutamate dehydrogenase of *Clostridium difficile* are highly functional for the detection of *Clostridium difficile* using stool samples.^25^ This was followed by our own study in which we derived a highly stable thrombin binding circular DNA aptamer, evolved directly in serum, that exhibits very high binding affinity, excellent anticoagulation activity and high stability in human serum.^16^ These studies highlight the advantage of circular aptamers and the benefit of performing circular aptamer selection directly in biological complex media. It is important to note that biological complex media like human serum represent molecular crowding environments where molecular species have higher viscosity and lower degrees of freedom than in aqueous buffer liquid. When adopted for aptamer selection, such media can minimize the entropic contribution and increase enthalpy contribution to molecular interactions, thus maximizing the opportunity of discovering high affinity aptamers with enhanced ability to resist matrix interference including nuclease degradation.^26–28^

**Fig. 1:**
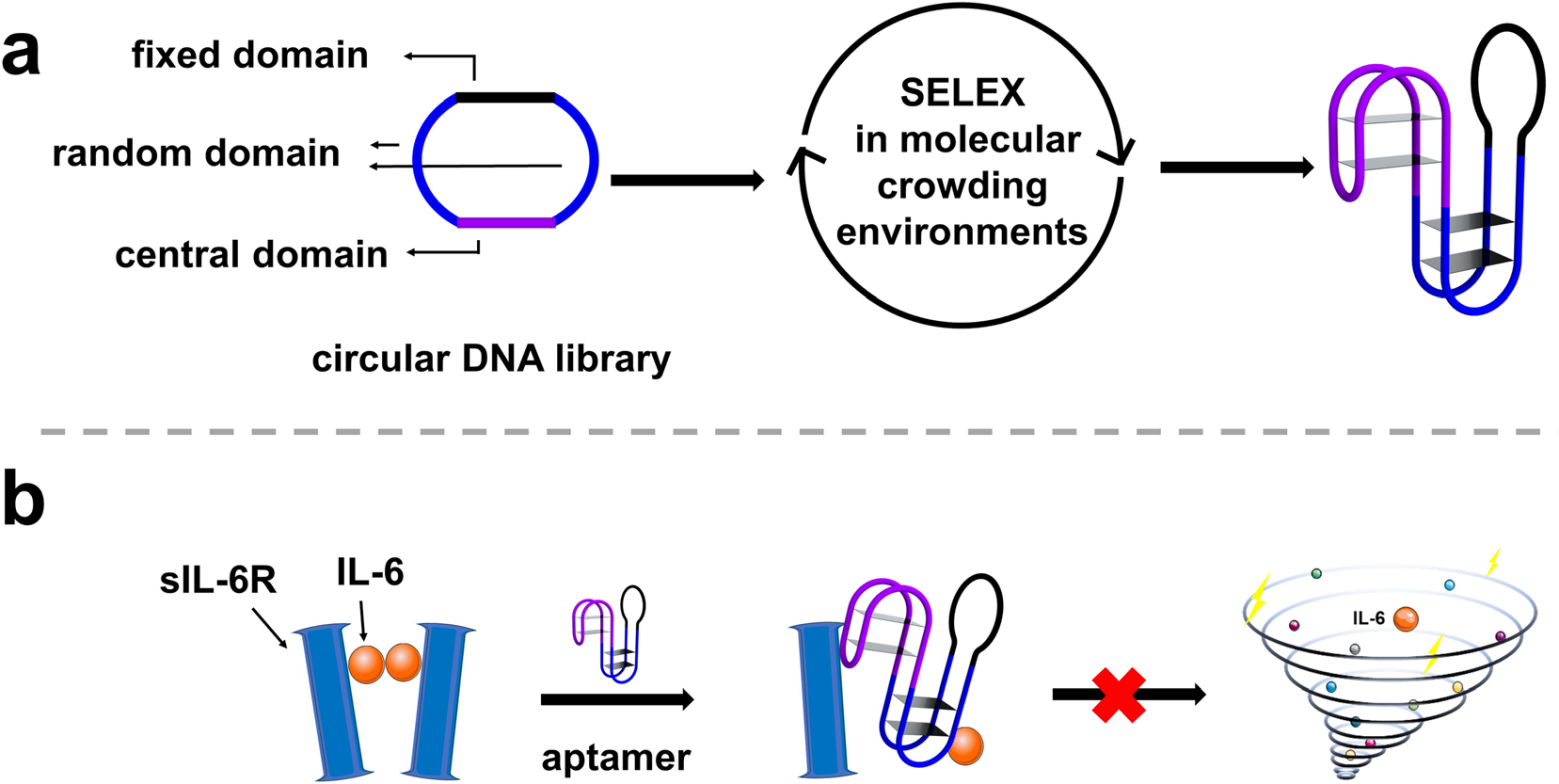
(a) Selection of IL-6/sIL-6R bispecific circular aptamer from a circular DNA library in molecular crowding environments. (b) Inhibition of cellular inflammation by the selected IL-6/sIL-6R bispecific circular aptamer.

Moreover, recent studies have shown that integrating two or more different biological recognition elements into one entity can enhance binding capabilities and expand recognition spectrum. Tan's group has developed dual-targeting circular aptamers and shown that they can recognize different leukemia cells with enhanced binding ability, thus making them more robust in cancer diagnosis and therapy.^29^ Han's group has designed bispecific aptamer chimeras that enable the degradation of targeted protein on cell membrane.^30^ Jia's group has created two aptamers into one circular aptamer, which showed significantly enhanced specificity and biological tolerance in serum.^31^ Although a linear aptamer can be redesigned into a bivalent circular aptamer, additional nucleotides will have to be carefully selected to construct circular aptamers to minimize the impact of circularization on aptamer activity.^32^ Given the importance of IL-6 and sIL-6R in a host of diseases, it is not surprising that DNA and RNA aptamers have already been developed to recognize IL-6 or sIL-6R.^5,6,33^ However, to date no effort has been made to develop aptamer-based therapeutic strategies that target both IL-6 and sIL-6R. In this work, we sought to investigate such a strategy as we hypothesize it is more effective than the single aptamer option. We set out to derive a circular DNA aptamer capable of binding both IL-6 and sIL-6R by performing aptamer selection with a circular DNA library in human serum (Fig. 1). We successfully obtained a bispecific circular aptamer, named CIL-6A6-1, that binds both IL-6 and sIL-6R with nanomolar affinities. More importantly, CIL-6A6-1 exhibits stronger ability to block IL-6/sIL-6R interaction and inhibit cell inflammation than aptamers, antibodies and small molecules that target IL-6 or sIL-6R separately for binding. These findings suggest that bispecific circular aptamers directly evolved in serum could be potentially developed into highly functional inhibitors for inflammation immunotherapy.

## Results and discussion

### Aptamer selection

Aptamer selection strategy was provided in Fig. S1a.† AIR-3A, an existing guanine-quartet (G4) forming RNA aptamer for sIL-6R (5′-GGGGAGGCUGUGGUGAGGG-3′)^33^ is converted into a DNA sequence (5′-GGGGAGGCTGTGGTGAGGG-3′) and incorporated as part of the sequence of the initial DNA library (Fig. S1b;† the sequences of all oligonucleotides are listed in Table S1†). We decided to use the DNA version of the aptamer because DNA is more stable, and the DNA aptamer converted from the RNA aptamer retained excellent binding affinity in our preliminary test (Table S2†). Specifically, the DNA library (5′-ATCTCGACTAN_20_GGGGAGGCTGTGG TGAGGG-N_20_-TGTCTCGGAT-3′) was designed to contain anti-sIL-6R DNA aptamer as the central element, flanked by two 20 nt random-sequence elements and two 10 nt fixed sequence elements. The circular DNA library was produced by end-to-end ligation of linear DNA library.

**Fig. 2:**
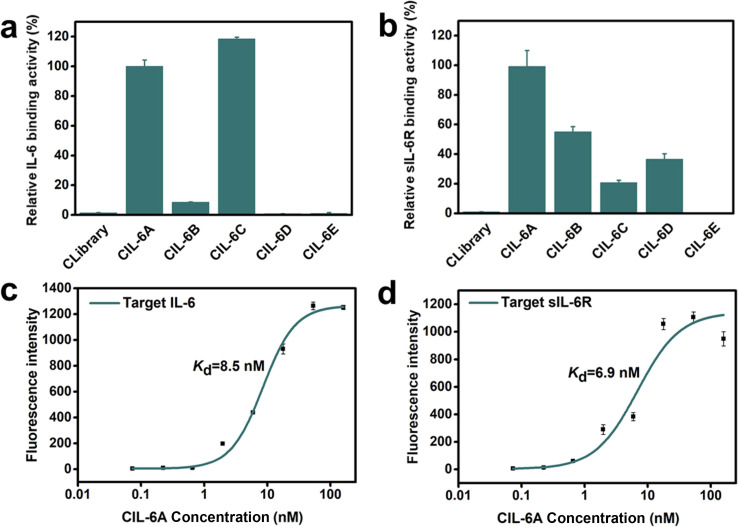
(a) Relative binding activity of the top five selected sequences for IL-6 (CIL-6A is taken as 100%). (b) Relative binding activity of the top five sequences for sIL-6R (CIL-6A is taken as 100%). CLibrary: the initial circular library, which was used as a control for both panels. Binding curves of CIL-6A against human (c) IL-6 and (d) sIL-6R.

Bare carboxylic acid magnetic beads (CAMBs) were first mixed with the circular DNA pool to remove beads-binding sequences (step 1, Fig. S1a†). The unbound DNA was incubated with the CAMBs coated with IL-6 (step 2). After the first round of selection, human serum (5%, 10%, 20%, 50% and 50% serum in rounds 2, 3, 4, 5 and 6, respectively) was added in step 2. The unbound DNA species were removed by washing (step 3). The bound DNA molecules were eluted by heating (step 4) and then amplified by a circle-to-circle amplification strategy we previously described (step 5).^34,35^ The enriched DNA pool was then used for the next round of selection. After six rounds of selection, the DNA pool was subjected to high-throughput DNA sequencing and many circular aptamers were discovered. SELEX experiments often lead to the enrichment of high-affinity aptamers that are ranked very high in the selected pools.^16^ For this consideration, we chose the top five ranked sequences in round 6 (Fig. S1c†) for affinity analysis using a real-time PCR-based pull-down assay (Fig. 2). Each circular aptamer was incubated with IL-6-coated CAMBs or sIL-6R-coated CAMBs, followed by elution of the bound aptamer and analysis by real time-PCR. Two of the five circular DNA molecules, CIL-6A and CIL-6C, showed much better binding affinity for IL-6 than the other three candidates (Fig. 2a). Four of the five candidates, CIL-6A-D, exhibited significant binding activity towards sIL-6R, but CIL-6E had weak binding activity for sIL-6R (Fig. 2b). Because CIL-6A demonstrated robust binding activity for both protein targets, it was chosen for the determination of the dissociation constant (*K*_d_) using the real time-PCR-based pull-down assay. CIL-6A was found to have *K*_d_ values of 8.5 nM and 6.9 nM respectively for IL-6 and sIL-6R (Fig. 2, panels c and d), indicating that it was an excellent bispecific aptamer and thus chosen for further investigation.

### Aptamer sequence characterization and optimization

**Fig. 3:**
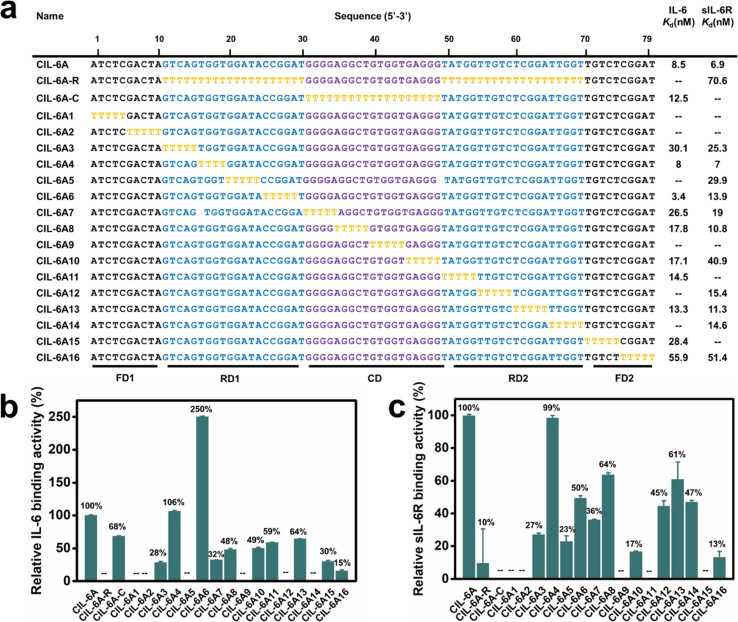
T-substitution study of CIL-6A. (a) For all the T-substitution sequences of CIL-6A, *K*_d_ against IL-6 and sIL-6R are reported. Relative binding activity of each T-substitution sequence with respect to CIL-6A (which is taken as 100%) towards human (b) IL-6 and (c) sIL-6R. The signal “—” denotes a sequence with no detectable binding affinity. Each data point represents the mean ± S. E. with three replicates.

The sequence of CIL-6A can be divided into 5 sequence elements as shown in Fig. 3: FD1 (fixed domain 1, A_1_-A_10_), RD1 (random domain 1, G_11_–T_30_), CD (central domain, G_31_–G_49_), RD2 (T_50_–T_69_), and FD2 (T_70_–T_79_). A mutant aptamer of CIL-6A, named CIL-6A-R, in which all nucleotides in the selected RD1 and RD2 sequence elements were mutated to dT-nucleotides, resulted in a complete loss of binding ability to IL-6 and significant reduction in binding ability to sIL-6R, suggesting that some or all the nucleotides in RD1 and RD2 were functionally important. Substituting all nucleotides within the CD domain with dT (CIL-6A-C; Fig. 3a) led to the complete loss of binding ability to sIL-6R but largely retained the affinity for IL-6; this was not surprising because this sequence element was used as the seeding aptamer for sIL-6R.

T-tract walking experiment was then performed to determine the importance of a group of consecutive nucleotides within each region of CIL-6A; in this experiment, each chosen nucleotide group was substituted with the same number of dT residues. Most of the mutant aptamers examined by the T-tract walking experiment with dT substitutions within FD1, RD1, CD, RD2 and FD2 showed no binding or significantly reduced binding ability for IL-6 and/or sIL-6R (CIL-6A1, 2, 3, 5, 7, 8, 9, 10, 11, 12, 13, 14, 15 and 16), suggesting that most of the nucleotides in these regions are involved in maintaining aptamer structural integrity and/or target binding. However, two mutants, CIL-6A4 (with 4 dT substitutions in RD1) and CIL-6A6 (with 5 dT substitutions also in RD1), were fully functional, indicating that the concerned nine nucleotides are functionally unimportant.

Interestingly, there are several GG elements in the selected random region. This pattern has been seen in many DNA sequences that have been shown to create G4 structures,^36–40^ pointing to the possibility that CIL-6A may adopt a G4 structure. This point will be investigated further later in this paper.

**Fig. 4:**
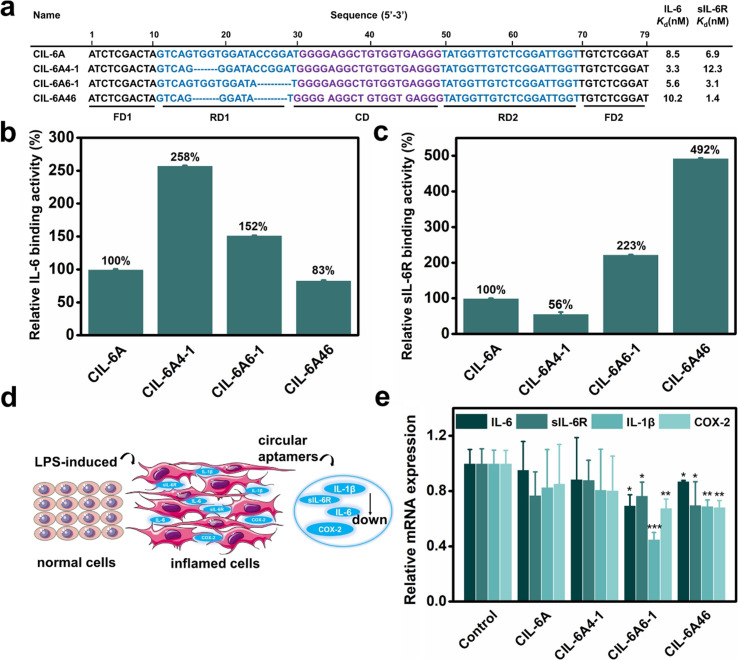
Sequence truncation study of CIL-6A. (a) Truncated sequences of CIL-6A and their *K*_d_ values against IL-6 and sIL-6R. (b and c) Relative binding activity of each truncated sequence of CIL-6A with respect to CIL-6A (taken as 100%) towards (b) IL-6 and (c) sIL-6R. Each data point represents the mean ± S. E. with three replicates. (d) Illustration of cytoprotection. (e) Inhibition effects on gene expression by 200 ng mL^−1^ CIL-6A, CIL-6A4-1, CIL-6A6-1 and CIL-6A46. Data are expressed as mean ± standard error of three experiments and each data point was compared with the control group (treatment only with 1 μg mL^−1^ LPS). *P < 0.05; **P < 0.01; ***P < 0.001 obtained using unpaired *t*-test followed by GraphPad Prism 6 test.

Based on the results from the T-walking experiment, we synthesized three truncated circular aptamers (CIL-6A4-1, CIL-6A6-1 and CIL-6A46) and tested their affinity to IL-6 and sIL-6R (Fig. 4, panels a–c). These three aptamers exhibited excellent affinity for both IL-6 and sIL-6R; in fact, CIL-6A6-1 represented a better aptamer than its parent aptamer as it produced smaller *K*_d_ values against both IL-6 and sIL-6R. It was previously reported that the inhibition efficiencies of the selected aptamers did not necessarily correlate with their binding affinities.^41^ The three truncated circular aptamers CIL-6A4-1, CIL-6A6-1, CIL-6A46, together with their parental aptamer CIL-6A, were then tested for their inhibitory effect on IL-6/sIL-6R induced inflammation (Fig. 4d). It is known that the IL-6/sIL-6R complex together with IL-1β exhibit a synergistic effect on inflammation; COX-2 is an important inflammatory marker, which can be used to reflect the degree of cell damage caused by inflammation.^42–45^ Based on these findings, the expression levels of IL-6, sIL-6, IL-1β and COX-2 were examined in this study to estimate the anti-inflammatory ability of the four circular aptamers CIL-6A4-1, CIL-6A6-1, CIL-6A 46, and CIL-6A (Fig. 4e). Interestingly, the inhibition efficiency of these aptamers correlated very well with their binding affinity. When treated with CIL-6A and CIL-6A4-1, the average expression levels of the four inflammatory factors were only slightly decreased. CIL-6A46 significantly down-regulated the expression levels of these four factors. CIL-6A6-1, which has the best binding affinity, produced strongest inhibition (*P < 0.05 for IL-6 and sIL-6R, **P < 0.01 for COX-2, and ***P < 0.001 for IL-1β). More importantly, the inhibitory effect of CIL-6A6-1 on the IL-6/sIL-6R induced inflammation was found to be dose-dependent (Fig. S2†). These results suggest that this bispecific circular aptamer may interact with IL-6 at the same site where sIL-6R binds.

### CIL-6A6-1 effectively inhibits inflammation

**Fig. 5:**
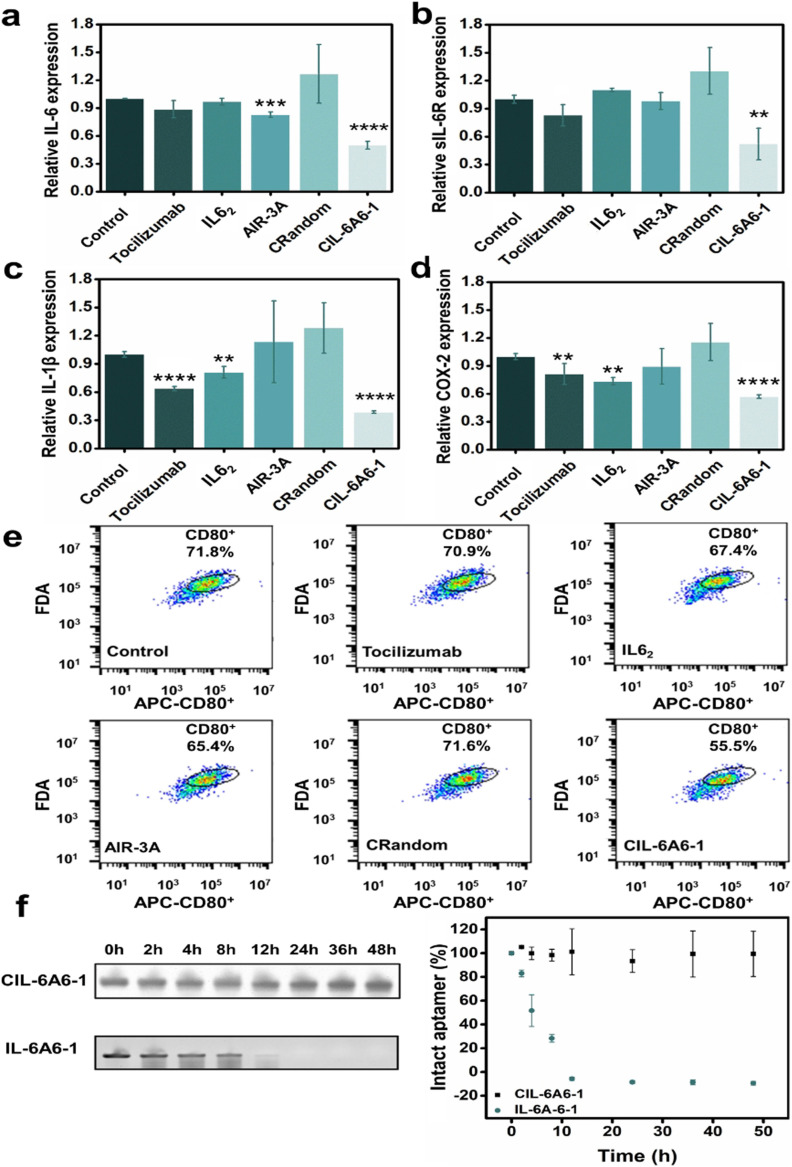
Comparison of gene expression inhibition efficiency on (a) IL-6, (b) sIL-6R, (c) IL-1β and (d) COX-2 by 2 μg mL^−1^ CIL-6A6-1, tocilizumab, IL-6 specific aptamer IL6_2_, sIL-6R-specific aptamer AIR-3A, a random circular sequence (CRandom) and no aptamer control (treatment only with 1 μg mL^−1^ LPS). Data are expressed as mean ± standard error of three experiments. Significance is denoted by asterisks in each figure: *P < 0.05; **P < 0.01; ***P < 0.001; ****P < 0.0001 (compared with the no aptamer control using unpaired *t*-test followed by GraphPad Prism 6 test). (e) Representative flow cytometry analysis of M1 macrophages (CD80^+^) in Raw264.7 cells with different treatments. The portion circled indicate macrophages that exhibit M1 polarization. The number in percentage in each plot represents the expression level of M1 macrophage (CD80^+^ cells). (f) Stability of CIL-6A6-1 and its linear form IL-6A6-1 in 50% serum. Left: gel images of CIL-6A6-1 and IL-6A6-1 after serum exposure for the indicated length of time. Right: graphical representation of gel results. The fraction of intact CIL-6A6-1 and IL-6A6-1 (relative to the zero time point control sample) was plotted as a function of time.

The inhibitory effect on the gene expression levels of the bispecific circular aptamer CIL-6A6-1 was further compared with tocilizumab, IL6_2_ (a known IL-6-specific DNA aptamer), AIR-3A (sIL-6R-specific aptamer) and a random circular sequence (CRandom). As shown in Fig. 5a–d, CIL-6A6-1 had the best inhibitory effect among these inhibitors, showing great inhibition efficiency on the gene expression levels of the four inflammatory cytokines (50% for IL-6, 52% for sIL-6R, 39% for IL-1β and 57% for COX-2). Subsequently, the inflammation inhibition ability of CIL-6A6-1 in macrophages was studied by flow cytometry analysis. Macrophages usually exist in the dormant M0 state, and when facing LPS or other stimulations, they switch to the activated M1 state and release inflammatory factors such IL-6, IL-1β and COX-2.^42^ We found that macrophages in M1 state being treated with 2 μg mL^−1^ CIL-6A6-1 and other inhibitors showed suppressed expression of CD80, and CIL-6A6-1 had the most significant inhibitory effect (Fig. 5e).

We also conducted two additional experiments to show the potency of the circular aptamer CIL-6A6-1. In the first experiment, we tested IL-6A6-1, the linear form of CIL-6A6-1, for the inhibition of IL-6/sIL-6R induced inflammation. The data presented in Fig. S3† shows that this linear aptamer produced no effect. This is justified as we further found that IL-6A6-1 did not bind either IL-6 or sIL-6R (Fig. S4†).

In the second experiment, we examined the inhibitory effect of combining IL6_2_ and AIR-3A – two separate aptamers that bind IL-6 and sIL-6R respectively – in a head-to-head comparison to the bispecific aptamer CIL-6A6-1. This test revealed that CIL-6A6-1 was superior to the two-aptamer mixture (Fig. S3†). The better inhibitory activity was also found to be consistent with higher binding affinity of CIL-6A6-1 for both protein targets over the two separate aptamers (Fig. S4†).

The results presented above clearly indicate that CIL-6A6-1 is a highly effective inhibitor of cellular inflammation, showing great potential as a therapeutic option for the treatment of various inflammatory diseases.

### High biostability in human serum

The biological stability of CIL-6A6-1 was further tested in a nuclease stability assay. It was found that even after incubation with 50% human serum for 48 hours, CIL-6A6-1 retained its sequence integrity, while the linear form IL-6A6-1 was easily degraded and had a half-life of only 4 h (Fig. 5f). These results clearly illustrate the superb capability of the selected circular aptamer against nuclease degradation, which is consistent with previous findings.^16,25^ The remarkable biological stability of this bispecific circular aptamer makes it highly desirable for therapeutic applications.

### Inhibition of IL-6/sIL-6R complex formation by three mechanisms employing CIL-6A6-1

To examine the potential utility of the bispecific aptamer as a mechanism-targeting therapeutic agent for IL-6/sIL-6R induced inflammation, CIL-6A6-1 was studied in three different assays, which we term prevention assay, competition assay and substitution assay, for its ability to inhibit the IL-6/sIL-6R interaction.

The prevention assay was designed to create the scenario of pre-blocking IL-6 or sIL-6R with CIL-6A6-1 as a neutralizing agent to prevent its natural partner (sIL-6R or IL-6) from binding, thereby testing CIL-6A6-1's utility as an inflammation prevention reagent. In this assay, IL-6 (or sIL-6R) was incubated with the aptamer pre-bound with sIL-6R (or IL-6) on beads. CIL-6A6-1 pre-bound with sIL-6R (top graphic, Fig. S5a†) inhibited the binding of IL-6 to sIL-6R by 94.1%; similarly, CIL-6A6-1 pre-bound with IL-6 (bottom graphic, Fig. S5a†) inhibited the binding of sIL-6R to IL-6 by 95%. These observations suggest that the aptamer is effective as a neutralizing agent to prevent the binding of IL-6 to sIL-6R.

The competition assay was designed to mimic the scenario of a developing inflammation where IL-6 and sIL-6R were being produced and a limited amount of the IL-6/sIL-6R complex was formed, and to test if the aptamer can inhibit the IL-6/sIL-6R complex formation. In this assay, the aptamer and IL-6 (or sIL-6R) were mixed with beads-bound sIL-6R (or beads-bound IL-6) at the same time. Under this setting, CIL-6A6-1 inhibited the binding of IL-6 to sIL-6R (top graphic, Fig. S5b†) by 53.8%, while it inhibited the binding of sIL-6R to IL-6 (bottom graphic, Fig. S5b†) by 78.8%. These results suggest that the aptamer could be potentially used as an effective inhibitor to prevent the IL-6/sIL-6R complex formation, thus suppressing a developing inflammation.

The substitution assay was designed to create the scenario of a well-developed inflammation where a large amount of the IL-6/sIL-6R complex has already formed, and to test the ability of CIL-6A6-1 to reduce the inflammation by displace IL-6 or sIL-6R from the IL-6/sIL-6R complex. In this assay, sIL-6R (top graphic, Fig. S5c†) or IL-6 (bottom graphic, Fig. S5c†) was first anchored on beads, then pre-bound with its natural binding partner, and subsequently mixed with the aptamer. Under these conditions, CIL-6A6-1 was found to be able displace 42% IL-6 bound to sIL-6R and 41.3% sIL-6R bound to IL-6 (Fig. S5c†). These discoveries suggest that the aptamer has the potential to treat IL-6/sIL-6R induced inflammation.

**Fig. 6:**
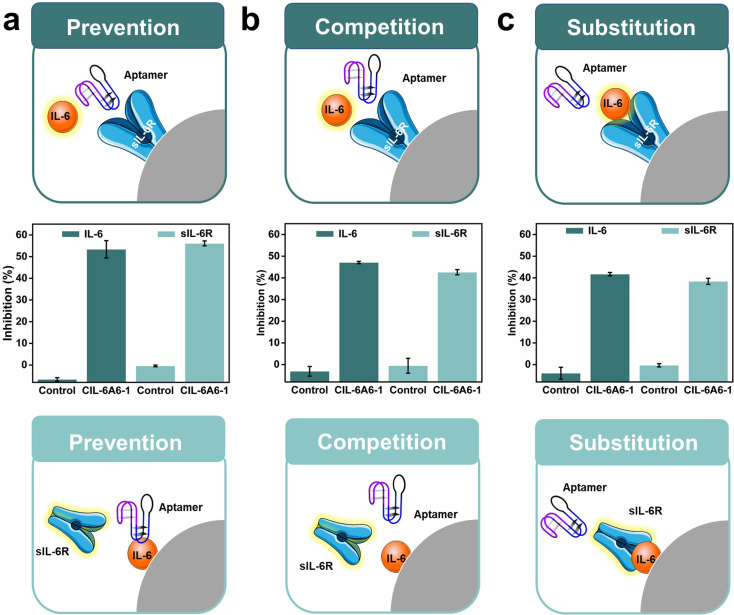
Assessment of efficiency of CIL-6A6-1 towards interfering with the interaction between IL-6 or sIL-6R in three different assays: (a) prevention assay, (b) competition assay and (c) substitution assay (based on fluorescently labeled IL-6 or sIL-6R). CRandom sequence was used as the control.

In order to further demonstrate that CIL-6A6-1 was an effective inhibitor for IL-6/sIL-6R induced inflammation, we investigated the effect of inhibition by CIL-6A6-1 on the binding either between fluorescently labelled IL-6 and unlabeled sIL-6R or between fluorescently labelled sIL-6R and unlabeled IL-6 in the prevention, competition and substitution assays (Fig. 6).

In the prevention assay, CIL-6A6-1 inhibited the binding of fluorescent IL-6 to aptamer-bound sIL-6R (top graphic, Fig. 6a) by 53.3%, and inhibited the binding of fluorescent sIL-6R to aptamer-bound IL-6 (bottom graphic, Fig. 6a) by 56.1%.

In the competition assay, CIL-6A6-1 inhibited the binding of fluorescent IL-6 to sIL-6R (top graphic, Fig. 6b) by 47.1%, and inhibited the binding of fluorescent sIL-6R to IL-6 (bottom graphic, Fig. 6b) by 42.5%. In the substitution assay, CIL-6A6-1 was able to displace 33.9% fluorescent IL-6 bound to sIL-6R (top graphic, Fig. 6c) and 38.2% fluorescent sIL-6R bound to IL-6 (bottom graphic, Fig. 6c).

Taken together, the data presented in Fig. 6 further corroborate the data presented in Fig. S5,† validating the potential of CIL-6A6-1 both as a preventative agent against and a treatment solution to the inflammation induced by IL-6/sIL-6R.

### A putative G-quadruplex structure of CIL-6A6-1

**Fig. 7:**
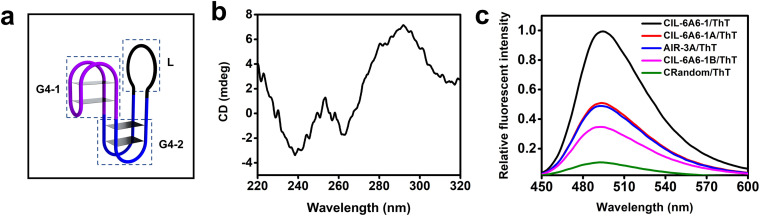
(a) The putative secondary structure of CIL-6A6-1. (b) Circular dichroism (CD) analysis of CIL-6A6-1. (c) Fluorescence emission spectra of ThT in the presence of CIL-6A6-1, CIL-6A6-1A, CIL-6A6-1B, AIR-3A and CRandom. The excitation wavelength was set at 425 nm. The sequences and putative secondary structures of CIL-6A6-1A and CIL-6A6-1B in relationship to CIL-6A6-1 are provided in Fig. S6.†

The G-rich nature of CIL-6A6-1 suggests that it may adopt a G4 structure. Previous study revealed that the GG elements within the constant region (the seeding aptamer region) create a G4 motif (G4-1, Fig. 7a).^33^ Searching the sequence in the selected random region led to the discovery of many other GG elements that may arranged into another G4 motif (G4-2, Fig. 7a). Circular dichroism (CD) was first employed to examine the existence of the G4 structure in the aptamer. The CD spectrum contains a negative peak close to 260 nm and a positive peak close to 290 nm, two features that are consistent with an antiparallel G4 (Fig. 7b). The additional negative peak around 240 nm may be caused by the influence of the flanking long sequences.^46–48^

The existence of G4 structure in CIL-6A6-1 was further confirmed with the binding assay using two fluorescent dyes and four control sequences. The two dyes were thioflavin T (ThT, a fluorescent dye that has been reported to bind G4 and produce enhanced fluorescence^49^) and *N*-methylmesoporphyrin IX (NMM, a fluorescent dye that has been demonstrated to specifically bind G4 and produce enhanced fluorescence^50,51^). The four control sequences were AIR-3A (known to contain a single 2-tiered G4), CIL-6A6-1A (a mutant of CIL-6A6-1 in which G4-1 – the first putative G4 motif – is replaced with a stem-loop structure; see Fig. S6†), and CIL-6A6-1B (another mutant of CIL-6A6-1 in which G4-1 and G4-2 – both putative G4 motifs – are replaced with a stem-loop structure; see Fig. S6†) and CRandom (a general DNA sequence control). As shown in Fig. 7c, the fluorescence intensity of the CIL-6A6-1/ThT mixture was the highest, followed by the AIR-3A/ThT mixture and the CIL-6A6-1A/ThT mixture, then by the CIL-6A6-1B/ThT mixture, and finally by the CRandom/ThT mixture. Similar observations were made with these sequences in the NMM experiment (Fig. S7†). Taken together, these results agree well with the proposed two G4 motifs in the structure of CIL-6A6-1.

We also performed the effect of Li^+^ on the stability of CIL-6A6-1 by examining the fluorescence of the CIL-6A6-1/NMM mixture in the absence of Li^+^ as well as in 50 and 100 mM of Li^+^, as it is known that Li^+^ destabilizes G4 structures.^52^ Not surprisingly, the fluorescent intensity of the solution was noticeably decreased with the addition of 50 mM Li^+^, and was further decreased in 100 nM Li^+^ (Fig. S8†).

The above experiments suggest that CIL-6A6-1 contains two independent G4 motifs in a single structure (G4-1 and G4-2), a structural arrangement that has never been observed with other aptamers. Given that this aptamer had evolved in human serum to recognize two different targets, it may have evolved to create an intricate structure to deliver three needed functions: a binding site for IL-6, a binding site for sIL-6R, and a property of being stable in human serum. Two different G4 elements along with the long loop element may represent an adequate solution for these challenges.

## Conclusions

In summary, we report the first effort to select circular DNA aptamers in human serum that can target two different protein molecules for binding. Specifically, we have isolated a serum-stable circular DNA aptamer, CIL-6A6-1, that can strongly bind both human IL-6 and sIL-6R. We further show that the aptamer has the potential to be used as a mechanism-targeting therapeutic agent for IL-6/sIL-6R induced inflammation through assays that simulate three different scenarios of IL-6/sIL-6R induced inflammation. Importantly, this bispecific aptamer is much more effective in inhibiting cellular inflammation than aptamers that bind IL-6 and sIL-6R alone as well as the monoclonal antibody tocilizumab targeting sIL-6R, suggesting that using an aptamer to target a cytokine and a receptor could very well represent a better therapeutic option. A remarkable feature of CIL-6A6-1 is that its putative structure contains two G-quadruplex motifs in a circular fold, which has not been observed previously for any aptamers. This unique structure fold may also contribute to its excellent functionality and high stability in human serum. The isolation of CIL-6A6-1 also highlights the advantage of selecting bispecific circular aptamers directly in crowded matrix, since this approach can produce high affinity recognition elements with improved functionality and biological stabilities in biological media. CIL-6A6-1 have properties well suited for anti-inflammation therapeutic challenge, including high affinity, good stability, minimum immunogenic risk and potent anti-inflammation ability.

## Data availability

The data supporting this article have been included as part of the ESI.†

## Author contributions

Lili Yao, Yu Mao, Yingfu Li and Lei Zheng conceived the study. Lili Yao, Yu Mao, Yingfu Li and Lei Zheng designed the experiments. Lili Yao, Lei Wang, Shuai Liu conducted the experiments. Lili Yao, Yu Mao, Yingfu Li and Lei Zheng analyzed and interpreted the data. Lili Yao, Yu Mao, Yingfu Li and Lei Zheng, wrote the manuscript, with input from the rest of the authors.

## Conflicts of interest

There are no conflicts to declare.

## Supplementary Material

SC-015-D4SC02183E-s001
