# Supplementary material for: Evolution of a bispecific G-quadruplex-forming circular aptamer to block IL-6/sIL-6R interaction for inflammation inhibition
Source: Chem Sci. 2024 Jul 17;15(32):13011–20. doi: 10.1039/d4sc02183e (PMC11323322; doi:10.1039/d4sc02183e)
Supplement: SC-015-D4SC02183E-s001 [file SC-015-D4SC02183E-s001.pdf]

Electronic Supplementary Material (ESI) for Chemical Science.
This journal is © The Royal Society of Chemistry 2024

Supporting Information

Evolution of a bispecific G-quadruplex-forming circular aptamer to block IL-6/sIL-6R interaction for inflammation inhibition

Lili Yao, Lei Wang, Shuai Liu, Hao Qu, Yu Mao*, Yingfu Li* and Lei Zheng*

Materials and methods

Oligonucleotides and other materials

All oligonucleotides (Table S1, Table S2 and Table S3) used in this work were synthesized and purified with HPLC in Sangon Biotech Company. The following reagents were purchased: recombinant human IL-6 from Solarbio; recombinant human sIL-6R receptor α from PEPRO TECH; Tocilizumab from Good Laboratory Practice bioscence; thioflavin T from MedChemExpress; N-methylmesoporphyrin Ⅸ (NMM) from SantaCruz; Monolith RED-HNS second generation protein labelling kit from Nanotemoer; T4 polynucleotide kinase (PNK), T4 DNA ligase, phi29 DNA polymerase, EcoRV, adenosine 5′-triphosphates (ATP) and deoxyribonucleoside 5′-triphosphates (dNTPs) from New England Biolabs; SGxcel Fast SYBR Mixture and 4S Red Plus from Sangon Biotech Company; PrimerScript™ RT regent Kit with gDNA Eraser (Perfect Real Time) and TB Green® Premix Ex Taq™ (Tli RNaseH Plus) from Takara; human serum from XinFan Biotech; CD80 APC from BD; Beaver Beads™COOH from BEAVER. Water was purified with a Milli-Q Synthesis from a Millipore system.

Instruments

The fluorescent images of gels were obtained using TOMOS Molecular Imager Gel Doc EX System. The intensity of each band was analyzed using Image J software. Real-Time PCR assay were performed on Bio-Rad CFX96 Real-Time PCR Detection System (Bio-Rad, Hercules, CA). Fluorescence measurements were carried out in a 96-well assay plate using a microplate reader (THERMO Varioskan Flash). Microscale thermophoresis were carried out on a Monolith NT.115 Pico (NanoTemper Technologies, Munich, Germany).

Library preparation

The DNA library contains a 19-nucleotide human sIL-6R binding aptamer flanked with two 20-nucleotide random domains and two constant domains at the 5′-end and 3′-end (note that the primer domain contains a recognition site for the restriction enzyme EcoRV), as shown in Figure S1b. The circular DNA library was prepared through template-assisted ligation with T4 DNA ligase. Briefly, linear DNA oligonucleotide was firstly phosphorylated as follows: a reaction mixture (60 µL) was made to contain 1.5 nmol linear oligonucleotide, 20 U PNK (U: unit), 1× PNK buffer (50 mM Tris-HCl, pH 7.6 at 25℃, 10 mM $MgCl_2$, 5 mM DTT, 0.1 mM spermidine) and 1 mM ATP. The mixture was incubated at 37℃ for 40 min, followed by heating at 90℃ for 5 min. The circularization reaction was conducted in a volume of 400 µl, produced by adding 306 µl of H_2O and 2 µl of a DNA template (LT1 or LT2, 100 µM) to the phosphorylation reaction mixture above. After heating at 90℃ for 3 min and cooling down at room temperature (RT) for 10 min, 40 µL of 10× T4 DNA ligase buffer (400 mM Tris-HCl, 100 mM $MgCl_2$, 100 mM DTT, 5 mM ATP, pH 7.8 at 25℃) and 2 µL of T4 DNA ligase (5 U/ µL) were added. This mixture was incubated in RT for 2 h follow by heating at 90℃ for 5 min. Lastly, the ligated circular DNA pool was concentrated by standard ethanol precipitation and purified by 10% dPAGE. The concentration of the circular DNA template was determined spectroscopically. The circular aptamer CIL-6A6-1 was further characterized by HPLC and mass spec analysis (Figure S9). The HPLC spectrum shows the presence of a single peak (7.305 min), indicating high purity of the circular products. The observed mass in the mass spectrum (23223.0) of circular products matches the theoretical mass of CIL-6A6-1.

Synthesis of IL-6/sIL-6R coated carboxylic acid magnetic beads (CAMBs)

Firstly, 500 µg CAMBs were washed three times using 100 mM MEST buffer (pH5.0, 0.05% Tween), after which 50 µL EDC (10 mg/mL) and 50 µL NHS (10 mg/mL) were added and incubated for 30 min at RT to activate the amino group on the CAMBs surface. Next, the tube was placed on a magnetic test tube holder and the supernatant was removed. After that, the activated CAMBs were incubated with 50 µL IL-6/sIL-6R (0.1 µg/µL) with rotation at 4℃ over night. Under the action of the magnet, the supernatant was removed. Subsequently, 100 µL PBST (10×PBS, pH7.2, 1% BSA) was added and incubated at RT with rotation for 1 h. Finally, the resulting products were washed three with 100 µL 1×PBS (pH7.2) and dispersed on 100 µL 1×PBS (pH7.2). The synthesized IL-6/sIL-6R coated CAMBs were stored at 4℃ until use.

Molecular crowding SELEX

Six SELEX rounds were carried out. For the first round, the circular DNA library (100 pmol), which was denatured at 95℃ for 5 min and then cooled on ice for 15 min, was incubated with CAMBs ($1×10^8$ beads / mL) with rotation for 1 h at room temperature in 100 µL of 1× binding buffer (1×PBSMT, pH 7.2) containing 137 mM NaCl, 2.68 mM KCl, 8.1mM Na_2HPO_4, 1.76 mM KH_2PO_4, 1 mM $MgCl_2$, and 0.025% Tween-20. The tube was then placed on a magnetic test tube holder, the supernatant was collected into a new tube. The collected circular oligonucleotides were incubated with CAMBs-bound human IL-6 ($1×10^8$ beads / mL) with rotation for 1 h at room temperature in 100 µL of 1× binding buffer. The tube was then placed on a magnetic test tube holder and the supernatant was removed. After that, the beads were washed three times

with 1× binding buffer (100 µL). Under the action of the magnet, protein-bound DNA molecules were eluted with 50 µL of ultrapure water at 95℃ for 15 min. After recovery by ethanol precipitation, the DNA was amplified by two rounds of rolling circle amplification (RCA), restricted digestion with EcoRV and circulation by DNA ligation. The RCA was typically performed in 50 µL of 1× RCA buffer (made from 10× stock, which is made of 330 mM Tris-acetate, pH 7.9 at 37℃, 100 mM magnesium acetate, 660 mM potassium acetate, 1% (v/v) Tween-20, 10 mM DTT) containing the DNA derived from last step, 2 µL LT1 (100 pmol), 1 mM dNTP. After heating at 90℃ for 3 min, the solution was cooled at room temperature for 10 min. Then, 0.5 µL of phi29 DNA polymerase (10 U/µL) was added, followed by incubation at 30℃ for 30 min before heated at 65℃ for 10 min to deactivate the polymerase. Subsequently, 2 µL of 500 µM LT2 was introduced to the RCA reaction mixture above. The mixture was heated at 90℃ for 3 min and cooled at RT for 10 min. Finally, 10 µL of 10× Fast Digestion Buffer (100 mM Tris-HCl, pH 8.0, 50 mM $MgCl_2$, 1 M NaCl, 1 mg/ml BSA) and 5 µL of Fast Digest EcoRV (unit size 400 reactions; the total volume is 400 µL) were added into above mixture and the final reaction volume was increased to 100 µL. The reaction mixture was then incubated at 37℃ for 8 h, and heated at 65℃ for 10 min to inactivate the restriction enzyme. The amplified monomerized RCA products were purified by 10% dPAGE. The DNA was then eluted from the gel and circularized into circular DNA template B (CDTB) for the second RCA reaction. The reaction condition was identical to the first RCA except for the replacement of LT1 with LT2. For the restriction digestion after RCA, LT2 was replaced with LT1. To create a molecular crowding environment, human serum was introduced into the binding buffer for rounds 2-6, and the amount of serum was progressively increased from 5% (round 2) to 10% (round 3), 20% (round 4), 50% (round 5) and 50% (round 6). The round-6 DNA pool was used for deep sequencing.

Sequencing protocol

The eluted DNA in round 6 was digested into linear DNA sequences as previously described. 2 µL of 0.05 µM digested DNA was amplified by two PCR steps. In PCR1, a 50 µL reaction mixture was prepared to contain the above DNA, 0.4 µM forward primer (F1) and 0.4 µM reverse primer (R1, their sequences are provided in Supplementary Table S1), 200 µM dNTP (dATP, dCTP, dGTP and dTTP), 1 × PCR buffer (75 mM Tris-HCl, pH 9.0, 2 mM $MgCl_2$, 50 mM KCl, 20 mM $(NH4)_2SO_4$) and 1.5 U Taq DNA polymerase. The DNA was amplified using the following thermal cycling steps: 94℃ for 3 min; 13 cycles at 94℃ (30 s), 45℃ (45 s), 72℃ (45 s) and 72℃ for 1 min. The PCR1 products were diluted 100 times, 2 µL of which was used as the template for PCR2 using the same primers and the same thermal cycling steps used above for PCR1. The DNA product produced by PCR2 was concentrated by standard ethanol precipitation and sent out for deep sequencing using an Illumina Miseq system at Sangon Biotech Company.

RT-PCR-based pull-down assay

Each aptamer candidate was incubated with IL-6-coated CAMBs or sIL-6R-coated CAMBs in 1× binding buffer. After incubation for 1 h, the tube was placed on a magnetic test tube holder and the supernatant was removed. After that, the beads were washed three times with 1× binding buffer. Protein-bound aptamers were eluted with ultrapure water at 95℃ for 15 min. Real-time PCR was used to quantify the eluted aptamers and the reaction conditions were using the following parameters: 30 cycles of denaturation at 94°C for 2 min, annealing at 45°C for 45 s, and elongation at 72°C for 45 s. The apparent dissociation constants (K_d) of all aptamer candidates were obtained by fitting the fluorescence value of the bound fraction of aptamers by varying total aptamer concentration, while the total target concentration is fixed. $y = D + (A – D)/(1 + (K/x)^B)$, where y = fluorescent intensity, x = aptamer concentration, A = maximum fluorescent intensity, B = the slope factor, K = K_d and D = minimal fluorescent intensity.

Microscale thermophoresis (MST)

IL-6 and sIL-6R proteins were labelled using Monolith RED-HNS second generation protein labelling kit. MST binding experiments were carried out using a constant concentration of labelled IL-6 and sIL-6R in 1 × binding buffer and varying concentrations of the aptamer. The experiments were carried out on a Monolith NT.115 Pico (NanoTemper Technologies, Munich, Germany) at 25℃ using standard capillaries. The recorded fluorescence was normalized and fitted using the K_d fit formula. Duplicate measurements were performed for each experimental setup. $y = D + (A – D)/(1 + (K/x)^B)$, where y = fluorescent intensity, x = aptamer concentration, A = maximum fluorescent intensity, B = the slope factor, K = K_d and D = minimal fluorescent intensity.

Prevention assay

In the IL-6 binding prevention assay, 200 nM CIL-6A6-1 was incubated with 200 nM sIL-6R-coated CAMBs at RT for 1 h so that CIL-6A6-1 would bind sIL-6R-coated CAMBs. The beads were washed twice using 1× binding buffer and resuspended in either 30 µL of 1× binding buffer containing 200 nM IL-6 (+target) or 30 µL of 1× binding buffer (-target). After incubation for 1 h, the beads were washed twice and the amount of CIL-6A6-1 remained on beads ($A_{+target}$ and $A_{-target}$) was quantified using the realtime-PCR-based pull-down assay described above. To correct for non-specific adsorption by beads, CIL-6A6-1 incubated with bare CAMBs was included to derive the amount of CIL-6A6-1 that was nonspecifically adsorbed by beads ($A_{background}$) The prevention efficiency (PE) is calculated as: $PE_{IL-6P} = [(A_{+target} - A_{background}) / (A_{-target} - A_{background})] \times 100\%$. The PE_{IL-6P} value was also derived for the control sequence (CRandom) using the same method. Three independent replicates were done for each sequence. The sIL-6R binding prevention assay was performed identically except for the use of IL-6-coated CAMBs and free sIL-6R. The prevention efficiency (PE) is calculated as: $PE_{sIL-6RP} = [(A_{+target} - A_{background}) / (A_{-target} - A_{background})] \times 100\%$. The $PE_{sIL-6RP}$ value was also derived for CRandom.

In the other binding prevention assay, IL-6 was fluorescently labelled. 2 μM CIL-6A6-1(+aptamer) or 1× binding buffer (-aptamer) was incubated with 200 nM sIL-6R-coated CAMBs at RT for 1 h. The beads are washed twice and resuspended in 1× binding buffer containing fluorophore labelled 200 nM IL-6. To correct for non-specific adsorption by beads, the bare beads incubated with fluorophore labelled IL-6 were included as the background signal ($F_{background}$). After the final incubation and washing, the amount of IL-6 remained on beads ($F_{+aptamer}$ and $F_{-aptamer}$) was quantified using the fluorescence spectrum analysis. The percentage of IL-6 binding prevention is calculated by the equation: $PE_{IL\text{-}6P} = [1- (F_{+aptamer} - F_{background}) / (F_{-aptamer} - F_{background})] \times 100\%$. The $PE_{IL\text{-}6P}$ value was also derived for the control sequence (CRandom) using the same method. Three independent replicates were done for each sequence. The sIL-6R binding prevention assay was performed identically except for the use of IL-6-coated CAMBs and fluorescently labelled sIL-6R. The prevention efficiency (PE) is calculated as: $PE_{sIL\text{-}6RP} = [1- (F_{+aptamer} - F_{background}) / (F_{-aptamer} - F_{background})] \times 100\%$. The $PE_{sIL\text{-}6RP}$ value was also derived for CRandom.

Competition assay

In the IL-6 binding competition assay, 200 nM CIL-6A6-1 was incubated with 200 nM sIL-6R-coated CAMBs in either 30 μL of 1× binding buffer containing 200 nM IL-6 (+target) or 30 μL of 1× binding buffer (-target) at RT for 1 h so that CIL-6A6-1 would bind sIL-6R-coated CAMBs with or without the competition of IL-6. After incubation for 1 h, the beads were washed twice and the amount of CIL-6A6-1 remained on beads ($A_{+target}$ and $A_{-target}$) was quantified using the realtime-PCR-based pull-down assay described above. To correct for non-specific adsorption by beads, CIL-6A6-1 incubated with bare CAMBs was included to derive the amount of CIL-6A6-1 that was nonspecifically adsorbed by beads ($A_{background}$). The competition efficiency (CE) is calculated as: $CE_{IL\text{-}6P} = [(A_{+target} - A_{background}) / (A_{-target} - A_{background})] \times 100\%$. The $CE_{IL\text{-}6P}$ value was also derived for the control sequence (CRandom) using the same method. Three independent replicates were done for each sequence. The sIL-6R binding competition assay was performed identically except for the use of IL-6-coated CAMBs and free sIL-6R. The competition efficiency (CE) is calculated as: $CE_{sIL\text{-}6RP} = [(A_{+target} - A_{background}) / (A_{-target} - A_{background})] \times 100\%$. The $CE_{sIL\text{-}6RP}$ value was also derived for CRandom.

In the other binding competition assay, 200 nM IL-6 was fluorescently labelled and incubated with 200 nM sIL-6R-coated CAMBs in either 1× binding buffer containing 2 μM CIL-6A6-1 (+aptamer) or 1× binding buffer (-aptamer) at RT for 1 h. After the final incubation and washing, the amount of IL-6 remained on beads ($F_{+aptamer}$ and $F_{-aptamer}$) was quantified using the fluorescence spectrum analysis. To correct for non-specific adsorption by beads, the bare beads incubated with fluorophore labelled IL-6 were included as the background signal ($F_{background}$). The percentage of IL-6 binding competition is calculated by the equation: $CE_{IL\text{-}6P} = [1- (F_{+aptamer} - F_{background}) / (F_{-aptamer} - F_{background})] \times 100\%$. The $CE_{IL\text{-}6P}$ value was also derived for the control sequence (CRandom) using the same method. Three independent replicates were done for each sequence. The sIL-6R binding competition assay was performed identically except for the use of IL-6-coated CAMBs and fluorescently labelled sIL-6R. The competition efficiency (CE) is calculated as: $CE_{sIL\text{-}6RP} = [1- (F_{+aptamer} - F_{background}) / (F_{-aptamer} - F_{background})] \times 100\%$. The $CE_{sIL\text{-}6RP}$ value was also derived for CRandom.

Substitution assay

In the IL-6 binding substitution assay, 200 nM IL-6 was incubated with 200 nM sIL-6R-coated CAMBs at RT for 1 h. The beads were washed twice using 1× binding buffer and resuspended in 30 μL of 1× binding buffer containing 200 nM CIL-6A6-1 so that CIL-6A6-1 would bind sIL-6R-coated CAMBs and substitute IL-6 from the IL-6/sIL-6R complex. After incubation for 1 h, the beads were washed twice and the amount of CIL-6A6-1 remained on beads (A) was quantified using the realtime-PCR-based pull-down assay described above. To correct for non-specific adsorption by beads, CIL-6A6-1 incubated with bare CAMBs was included to derive the amount of CIL-6A6-1 that was nonspecifically adsorbed by beads ($A_{background}$). Equal amount of CIL-6A6-1 originally added was also quantified by realtime-PCR (A_{sum}). The substitution efficiency (SE) is calculated as: $SE_{IL\text{-}6P} = [(A - A_{background}) / (A_{sum})] \times 100\%$. The $SE_{IL\text{-}6P}$ value was also derived for the control sequence (CRandom) using the same method. Three independent replicates were done for each sequence. The sIL-6R binding substitution assay was performed identically except for the use of IL-6-coated CAMBs and free sIL-6R. The substitution efficiency (SE) is calculated as: $SE_{sIL\text{-}6RP} = [(A - A_{background}) / (A_{sum})] \times 100\%$. The $SE_{sIL\text{-}6RP}$ value was also derived for CRandom.

In the other binding substitution assay, 200 nM fluorophore labelled IL-6 was incubated with 200 nM sIL-6R-coated CAMBs at RT for 1 h. The beads are washed twice and resuspended in 1× binding buffer containing 2 μM CIL-6A6-1. After incubation for 1 h, the beads were washed twice and the amount of IL-6 remained on beads (F) was quantified using the fluorescence spectrum analysis. To correct for non-specific adsorption by beads, the bare beads incubated with fluorophore labelled IL-6 were included as the background signal ($F_{background}$). Equal amount of IL-6 originally added was also quantified by fluorescence spectrum analysis (F_{sum}). The substitution efficiency (SE) is calculated as: $SE_{IL\text{-}6P} = [1- (F - F_{background}) / (F_{sum})] \times 100\%$. The $SE_{IL\text{-}6P}$ value was also derived for the control sequence (CRandom) using the same method. Three independent replicates were done for each sequence. The sIL-6R binding substitution assay was performed identically except for the use of IL-6-coated CAMBs and free sIL-6R. The substitution efficiency (SE) is calculated as: $SE_{sIL\text{-}6RP} = [1- (F - F_{background}) / (F_{sum})] \times 100\%$. The $SE_{sIL\text{-}6RP}$ value was also derived for CRandom.

Stability assay

6 μM circular anti-human IL-6 aptamer and the corresponding linear aptamer were dissolved in 1× binding buffer and incubated in 50% human serum at 37℃ for 0, 2, 4, 8, 12, 24, 36, 48h. At established time points, 20 μL samples were heated at 95°C for 10 min to denature enzymes

and then stored at -20°C until all samples were collected. Prior to electrophoresis analysis, samples were prepared for electrophoresis by addition of 2 × denaturing gel loading buffer to 1× final concentration. Then, samples were loaded and 10% 1× TBE denaturing PAGE was run at 110 V for 30 min at 25°C. The gel was visualized using a TOMOS Molecular Imager Gel Doc EX System and the bands were quantified using ImageJ software. The half-life value was determined by quantifying the time required for the aptamer concentration to degrade to 50% of its initial level.

RAW264.7 cell culture

The murine macrophage cell line, RAW264.7, was purchased from COBIOER in Nanjing. RAW264.7 cells were cultured at 37℃ in a 5% CO_2 incubator in RPMI-1640 medium supplemented with 5% heat-inactivated fetal bovine serum, 100 U/ml penicillin and 100 µg/ml streptomycin.

RNA extraction, cDNA synthesis, and real-time quantitative PCR assay

RAW264.7 cells were seeded in a 24-well culture plate at 10^5 cells / well and incubated overnight at 37℃ with 5% CO_2. Cells were activated with 1 µg/ml LPS and simultaneously treated with various concentration of IL-6A6-1 circular aptamer (CIL-6A6-1) (2 µg/ml, 200 ng/mL, 20 ng/mL), 200 ng/mL CIL-6A, CIL-6A4-1, CIL-6A6-1 and CIL-6A46 or some other positive control groups (2 µg/ml sIL-6R-specific RNA aptamer AIR-3A, IL-6-specific aptamer $IL6_2$, random circular aptamer CRandom and Tocilizumab) for 12 h. For total RNA extraction, the adherent cells were lysed with Trizol reagent (Sangon Biotech, Shanghai, China). RNA from each sample was converted to cDNA by Takara PrimeScript RT reagent Kit (Takara, Dalian, China) according to the manufacturer’s instructions. The qPCR reaction was performed by Bio-Rad CFX96 Real-Time PCR Detection System (Bio-Rad, Hercules, CA) and the reaction conditions were using the following parameters: 30 cycles of reactions of denaturation at 95°C for 2 min, annealing at 55°C for 30 s and elongation at 72°C for 45 s. The expression of target genes, including IL-6, sIL-6, IL-1β, and COX-2 were evaluated in this study. Primer sets of target genes and the actin gene (β-Actin) for qPCR are shown in Supplementary Table S3.

Flow cytometry

RAW264.7 cells were seeded in a 6-well culture plate at 10^6 cells / well and incubated overnight at 37℃ with 5% CO_2. After incubation, cells were activated with 1 µg/ml LPS and simultaneously treated with 2 µg/ml of IL-6A6-1 circular aptamer and some other positive control groups (2 µg/ml sIL-6R-specific RNA aptamer AIR-3A, IL-6-specific aptamer $IL6_2$, CRandom and Tocilizumab) for 12 h. At the indicated time, the anti-CD16/32 antibody (Biolegend 101319, California, United States) blocked the Fc receptor on the surface of RAW264.7 cells and incubated on ice for 10 min. Then, macrophages were harvested and stained with the fluorescein diacetate (FDA, live cells stain) and APC-conjugated anti-mouse CD80 in the dark for 30 min at 4°C. After that, the labeled cells were analyzed using a flow cytometer (Beckman Coulter Navios, San Jose, CA, USA). Data were analyzed with Flow Jo 10 software.

Fluorescence measurements of G-quadruplex–ligand interaction

Thioflavin T (ThT, 10 µM) and the relevant circular DNA (300 nM) were incubated in the 1× binding buffer for 30 min. Fluorescence measurements were carried out in a 96-well assay plate using a microplate reader (THERMO Varioskan Flash) at room temperature. The excitation wavelength of the solution was set at 425 nm, and the emission spectra were collected from 450 to 600nm with a step of 2 nm.

N-methylmesoporphyrin Ⅸ (NMM, 5 µM) and the relevant circular DNA (300 nM) were incubated in the 1× binding buffer for 30 min. Fluorescence measurements were carried out in a 96-well assay plate using a microplate reader (THERMO Varioskan Flash) at room temperature. The excitation wavelength of the solution was set at 399 nm, and the emission spectra were collected from 550 to 780nm with a step of 2 nm.

Circular dichroism analysis

Circular dichroism (CD) studies were carried out using JASCO J-1700 instrument according to the manufacturer’s instructions. All constructs were scanned from 320 to 220 nm at 16 µM DNA in a 0.1-cm quartz cuvette. All DNA samples were incubated for 30 min at room temperature in the 1× binding buffer before scanning.

Supplement Tables

Table S1. Sequences of oligonucleotides used in this work

Name of DNA oligonucleotide	Sequence (5'-3')
DNA library (Lib)	ATCTC GACTA N20 GGGG AGGCT GTGGT GAGGG N20 TGTCT CGGAT
IL6$_2$	CTTCC AACGC TCGTA TTGTC AGTCT TTAGT
AIR-3A	GGGGA GGCUG UGGUG AGGG
AIR-3A DNA counterpart	GGGGA GGCTG TGGTG AGGG
CRandom	TAGTC GAATC TTCCC GTTCT GTATA AATTT ACCTG GTTAC TTAAG CCCTT ATTCC TCTGT TACGT TTCCT AGCTG ACGC
CRandom Template1	GATTC GACTA GCGTC AGCTA
CRandomF1	GCCTC AACTT GCGTC AGCTA
CRandomF2	GGCAC TGTCA TAGTC GAATC
CIL-6A	ATCTC GACTA GTCAG TGGT GGATA CCGGA TGGGG AGGCT GTGGT GAGGG TATGG TTGTC TCGGA TTGGT TGTCT CGGAT
CIL-6A-R	ATCTC GACTA TTTTT TTTT TTTTT TTTTT TGGGG AGGCT GTGGT GAGGG TTTTT TTTTT TTTTT TTTTT TGTCT CGGAT
CIL-6A-C	ATCTC GACTA GTCAG TGGT GGATA CCGGA TTTTT TTTTT TTTTT TTTTT TATGG TTGTC TCGGA TTGGT TGTCT CGGAT
CIL-6A1	TTTTT GACTA GTCAG TGGT GGATA CCGGA TGGGG AGGCT GTGGT GAGGG TATGG TTGTC TCGGA TTGGT TGTCT CGGAT
CIL-6A2	ATCTC TTTTT GTCAG TGGT GGATA CCGGA TGGGG AGGCT GTGGT GAGGG TATGG TTGTC TCGGA TTGGT TGTCT CGGAT
CIL-6A3	ATCTC GACTA TTTTT TGGT GGATA CCGGA TGGGG AGGCT GTGGT GAGGG TATGG TTGTC TCGGA TTGGT TGTCT CGGAT
CIL-6A4	ATCTC GACTA GTCAG TTTT GGATA CCGGA TGGGG AGGCT GTGGT GAGGG TATGG TTGTC TCGGA TTGGT TGTCT CGGAT
CIL-6A5	ATCTC GACTA GTCAG TGGT TTTTT CCGGA TGGGG AGGCT GTGGT GAGGG TATGG TTGTC TCGGA TTGGT TGTCT CGGAT
CIL-6A6	ATCTC GACTA GTCAG TGGT GGATA TTTTT TGGGG AGGCT GTGGT GAGGG TATGG TTGTC TCGGA TTGGT TGTCT CGGAT
CIL-6A7	ATCTC GACTA GTCAG TGGT GGATA CCGGA TTTTT AGGCT GTGGT GAGGG TATGG TTGTC TCGGA TTGGT TGTCT CGGAT
CIL-6A8	ATCTC GACTA GTCAG TGGT GGATA CCGGA TGGGG TTTTT GTGGT GAGGG TATGG TTGTC TCGGA TTGGT TGTCT CGGAT
CIL-6A9	ATCTC GACTA GTCAG TGGT GGATA CCGGA TGGGG AGGCT TTTTT GAGGG TATGG TTGTC TCGGA TTGGT TGTCT CGGAT
CIL-6A10	ATCTC GACTA GTCAG TGGT GGATA CCGGA TGGGG AGGCT GTGGT TTTTT TATGG TTGTC TCGGA TTGGT TGTCT CGGAT
CIL-6A11	ATCTC GACTA GTCAG TGGT GGATA CCGGA TGGGG AGGCT GTGGT GAGGG TTTTT TTGTC TCGGA TTGGT TGTCT CGGAT
CIL-6A12	ATCTC GACTA GTCAG TGGT GGATA CCGGA TGGGG AGGCT GTGGT GAGGG TATGG TTTTT TCGGA TTGGT TGTCT CGGAT
CIL-6A13	ATCTC GACTA GTCAG TGGT GGATA CCGGA TGGGG AGGCT GTGGT GAGGG TATGG TTGTC TTTTT TTGGT TGTCT CGGAT
CIL-6A14	ATCTC GACTA GTCAG TGGT GGATA CCGGA TGGGG AGGCT GTGGT GAGGG TATGG TTGTC TCGGA TTTTT TGTCT CGGAT
CIL-6A15	ATCTC GACTA GTCAG TGGT GGATA CCGGA TGGGG AGGCT GTGGT GAGGG TATGG TTGTC TCGGA TTGGT TTTTT CGGAT
CIL-6A16	ATCTC GACTA GTCAG TGGT GGATA CCGGA TGGGG AGGCT GTGGT GAGGG TATGG TTGTC TCGGA TTGGT TGTCT TTTTT
CIL-6A4-1	ATCTC GACTA GTCAG GGATA CCGGA TGGGG AGGCT GTGGT GAGGG TATGG TTGTC TCGGA TTGGT TGTCT CGGAT
CIL-6A6-1	ATCTC GACTA GTCAG TGGT GGATA TGGGG AGGCT GTGGT GAGGG TATGG TTGTC TCGGA TTGGT TGTCT CGGAT
CIL-6A6-1A	ATCTC GACTA GTCAG TGGT GGATA TGTGC ACACT GTTGT GAACG TATGG TTGTC TCGGA TTGGT TGTCT CGGAT
CIL-6A6-1B	ATCTC GACTA GTCAG TATT CTATA TGTGC ACACT GTTGT GAACG TATAG TTGTC TCATA TTGGT TGTCT CGGAT
CIL-6A46	ATCTC GACTA GTCAG GGATA TGGGG AGGCT GTGGT GAGGG TATGG TTGTC TCGGA TTGGT TGTCT CGGAT
Ligation template1 (LT1)	TAGTC GAGAT ATCCG AGACA

Ligation template1 (LT2)	TGTCT CGGAT ATCTC GACTA
Ligation template1 (CIL-6A1)	TAGTC AAAAA ATCCG AGACA
Ligation template1 (CIL-6A2)	AAAAA GAGAT ATCCG AGACA
Ligation template1 (CIL-6A15)	TAGTC GAGAT ATCCG AAAAA
Ligation template1 (CIL-6A16)	TAGTC GAGAT AAAAA AGACA
Forward primer (F1)	GCCTC AACTT ATCCG AGACA
Reverse primer (R1)	GGCAC TGTCA ATCTC GACTA
Reverse primer (CIL-6A1)	GGCAC TGTCA TTTTT GACTA
Reverse primer (CIL-6A2)	GGCAC TGTCA ATCTC TTTTT
Forward primer (CIL-6A15)	GCCTC AACTT ATCCG AAAAA
Forward primer (CIL-6A16)	GCCTC AACTT AAAAA AGACA

Table S2. The K_d values of AIR-3A and its DNA counterpart against sIL-6R measured using MST

Name of DNA oligonucleotide	Sequence (5'-3')	K_d
AIR-3A	GGGGA GGCUG UGGUG AGGG	67.5 nM
AIR-3A DNA counterpart	GGGGA GGCTG TGGTG AGGG	131.2 nM

Table S3. Primer for RT PCR used in this work

Genes	Forward primer (5'-3')	Reverse primer (5'-3')
β-actin-	GGCCC AGAGC AAGAG AGGTA	GCTGG GGTGT TGAAG GTCTC
IL-6	TGGAG TCACA GAAGG AGTGG CTAAG	TCTGA CCACA GTGAG GAATG TCCAC
sIL-6R	GGTTG CTTCT CTGTG TCTTC ATCA	AAGTA GTCTC TGTTG CTGTT GTCA
IL-1β	TCTTT TGGGG TCCGT CAACT	GCAAC TGTTC CTGAA CTCAA CT
COX-2	GGAGA GACTA TCAAG ATAGT GATC	ATGGT CAGTA GACTT TTACA GCTC

Supplement Figures

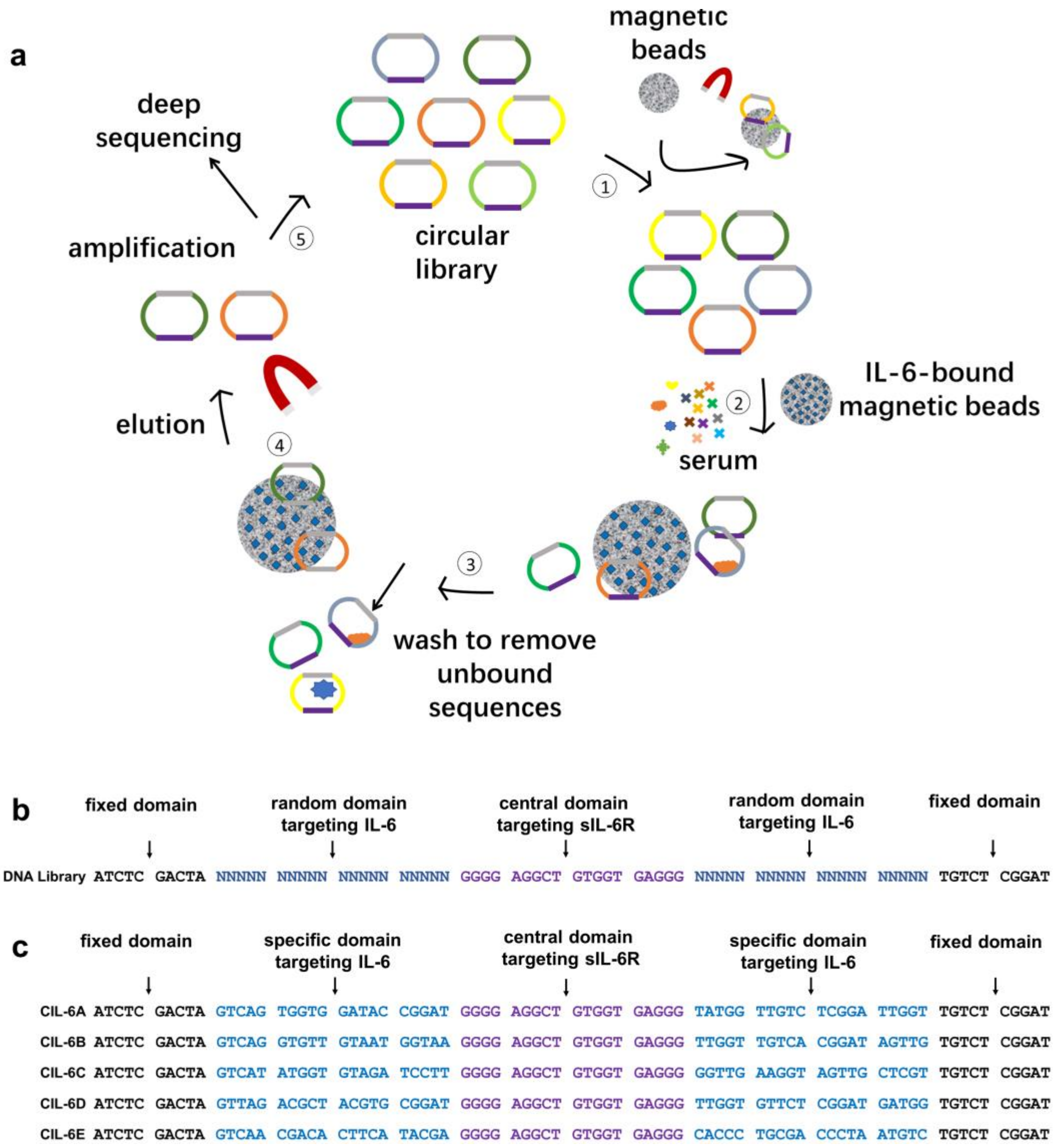

Figure S1. (a) Molecular crowding directed selection of bispecific circular aptamers for IL-6/sIL-6R. (b) The sequence of the original DNA library. (c) The sequences of top 5 aptamer candidates.

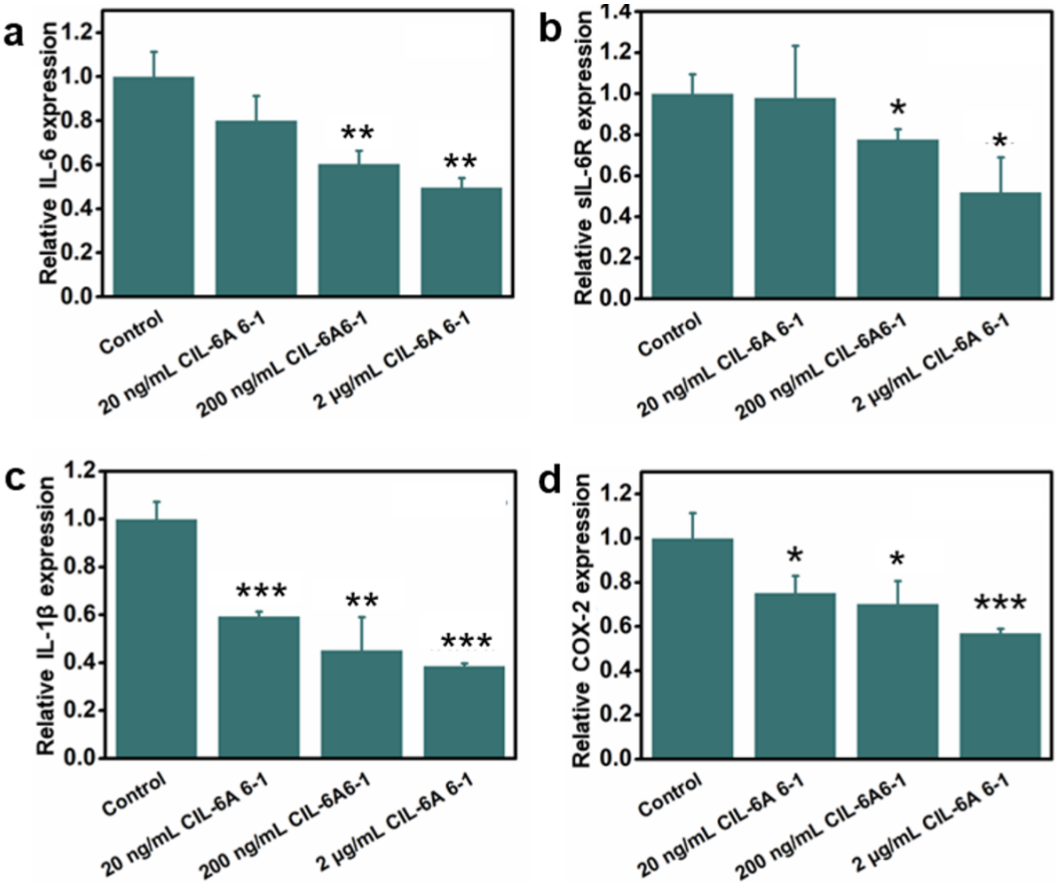

Figure S2. Dose-dependent inhibitory effect of CIL-6A6-1 on the IL-6/sIL-6R induced inflammation. CIL-6A6-1 was tested at different concentrations (20 ng/mL, 200 ng/mL, and 2 μg/mL) for suppression of gene expression of (a) IL-6, (b) sIL-6R, (c) IL-1β and (d) COX-2 in RAW 264.7 cells. Data are expressed as mean ± standard error of three experiments. Significance is denoted by asterisks in each figure: *P< 0.05; **P< 0.01; ***P< 0.001 in comparison to the control group (only treated with 1 μg/ml LPS) using unpaired t-test followed by GraphPad Prism 6 test).

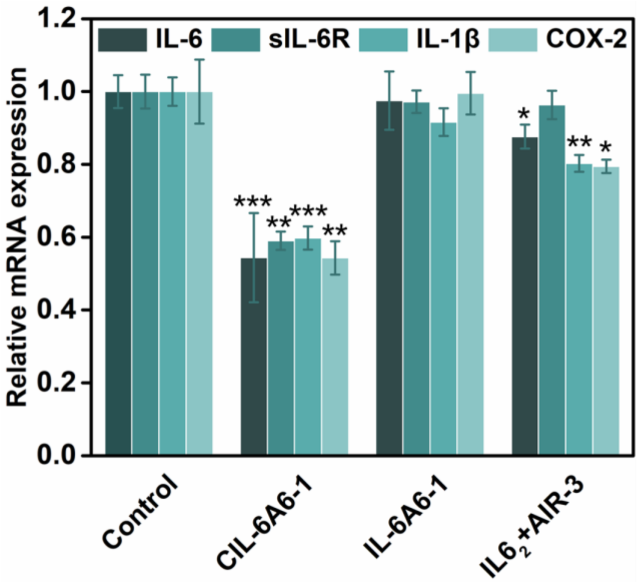

Figure S3. Comparison of gene expression inhibition efficiency of 2 μg/mL CIL-6A6-1, IL-6A6-1 and IL6$_2$+AIR-3A (the mixture of IL-6-specific aptamer IL6$_2$ and sIL-6R-specific aptamer AIR-3A) with the control (treatment only with 1 μg/mL LPS). The data are expressed as mean ± standard error of three experiments. Significance is denoted by asterisks in each figure: *P< 0.05; **P< 0.01; ***P< 0.001 (compared with the control group (only treated with 1 μg/ml LPS) using unpaired t-test using GraphPad Prism 6).

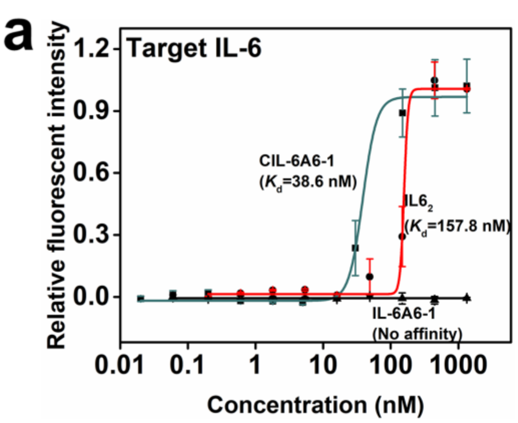

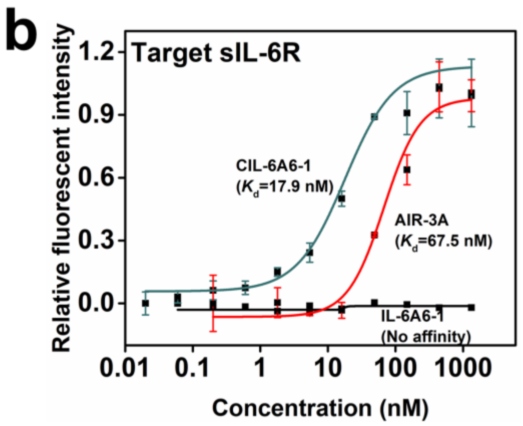

Figure S4. Binding curves of CIL-6A6-1, IL-6A6-1, IL6$_2$ and AIR-3A against human IL-6 and sIL-6R measured by MST assay.

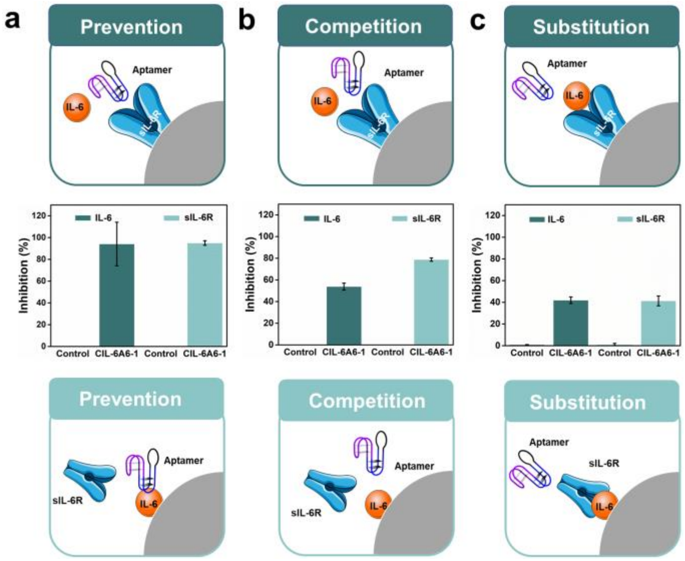

Figure S5. Assessment of RT-PCR-based efficiency of CIL-6A6-1 towards interfering the interaction between IL-6 or sIL-6R in three different assays: a) prevention assay, b) competition assay and c) substitution assay. CRandom sequence was used as control.

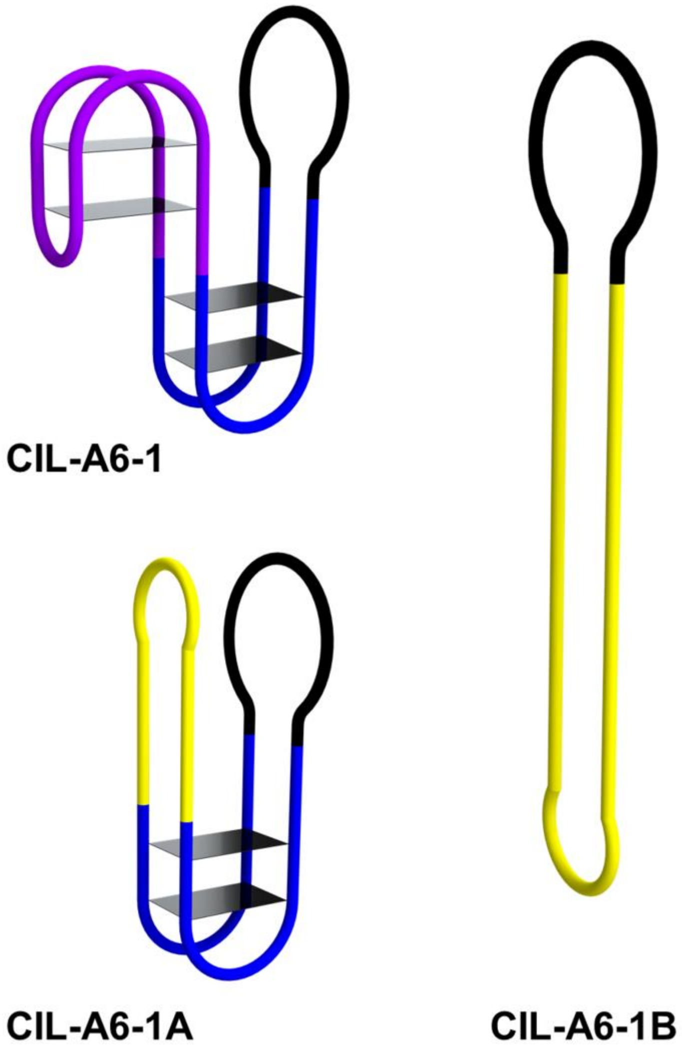

Figure S6. The putative secondary structures of CIL-6A6-1, CIL-6A6-1A and CIL-6A6-1B.

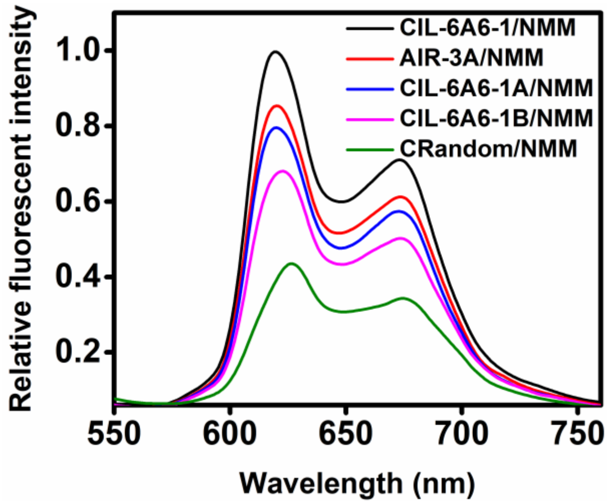

Figure S7. Fluorescence emission spectra of N-methylmesoporphyrin IX (NMM) in the presence of CIL-6A6-1, CIL-6A6-1A, CIL-6A6-1B, AIR-3A and CRandom. The excitation wavelength was set at 399 nm. The sequences and putative secondary structures of CIL-6A6-1A and CIL-6A6-1B in relationship to CIL-6A6-1 are provided in Figure S6.

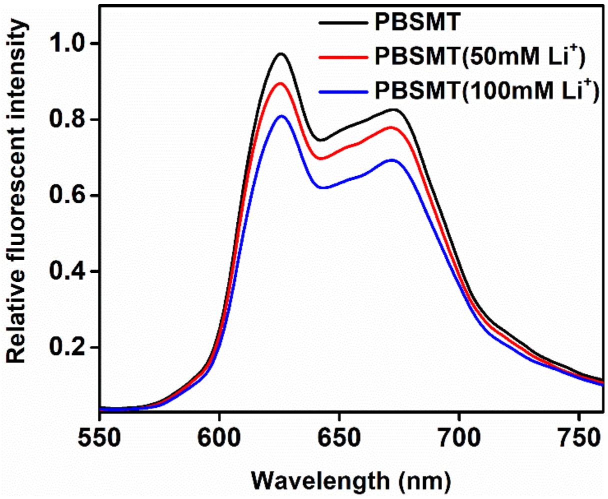

Figure S8. Fluorescence emission spectra of N-methylmesoporphyrin Ⅸ (NMM) in the presence of CIL-6A6-1 at different Li^+ concentrations. The excitation wavelength was set at 399 nm.

a

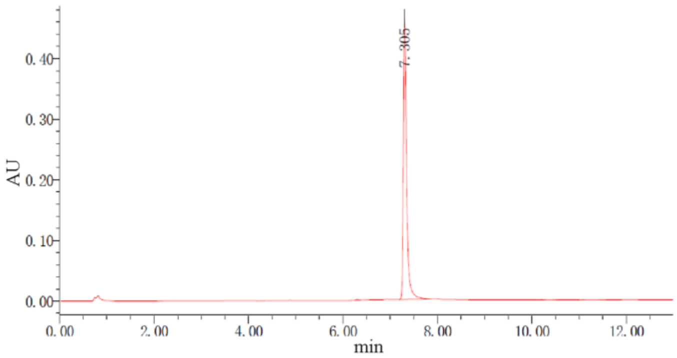

b

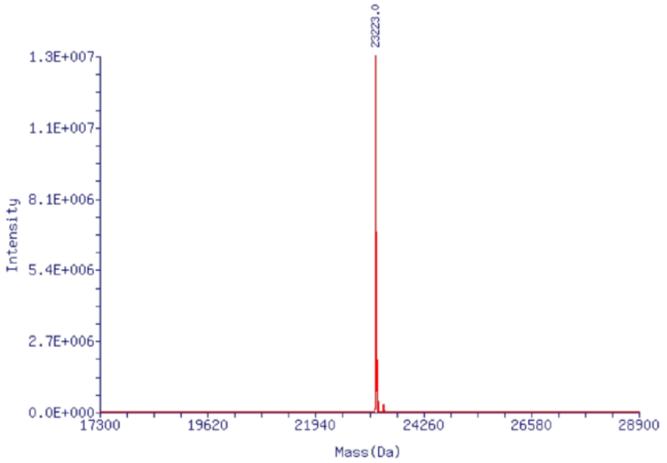

Figure S9. (a) HPLC and (b) mass spectra of CIL-6A6-1.
